# Immune Cell-Related Genes in Juvenile Idiopathic Arthritis Identified Using Transcriptomic and Single-Cell Sequencing Data

**DOI:** 10.3390/ijms241310619

**Published:** 2023-06-25

**Authors:** Wenbo Zhang, Zhe Cai, Dandan Liang, Jiaochan Han, Ping Wu, Jiayi Shan, Guangxun Meng, Huasong Zeng

**Affiliations:** 1 The Joint Center for Infection and Immunity, Guangzhou Institute of Pediatrics, Guangzhou Women and Children’s Medical Center, Guangzhou 510623, China; 20193101009@stu.gzucm.edu.cn; 2 The Joint Center for Infection and Immunity, CAS Key Laboratory of Molecular Virology & Immunology, Chinese Academy of Sciences, Shanghai 200031, China; 3 The First Clinical Medical School, Guangzhou University of Chinese Medicine, Guangzhou 510006, China; 4 Department of Allergy, Immunology and Rheumatology, Guangzhou Women and Children’s Medical Center, Guangzhou Medical University, Guangzhou 510623, China; 5 Guangzhou Institute of Pediatrics, Guangzhou Women and Children’s Medical Center, Guangzhou 510623, China; 6 The Center for Microbes, Development and Health, CAS Key Laboratory of Molecular Virology & Immunology, University of Chinese Academy of Sciences, Shanghai 200031, China

**Keywords:** juvenile idiopathic arthritis, WGCNA, protein–protein interaction, machine learning analysis, single-cell RNA sequencing

## Abstract

Juvenile idiopathic arthritis (JIA) is the most common chronic rheumatic disease in children. The heterogeneity of the disease can be investigated via single-cell RNA sequencing (scRNA-seq) for its gap in the literature. Firstly, five types of immune cells (plasma cells, naive CD4 T cells, memory-activated CD4 T cells, eosinophils, and neutrophils) were significantly different between normal control (NC) and JIA samples. WGCNA was performed to identify genes that exhibited the highest correlation to differential immune cells. Then, 168 differentially expressed immune cell-related genes (DE-ICRGs) were identified by overlapping 13,706 genes identified by WGCNA and 286 differentially expressed genes (DEGs) between JIA and NC specimens. Next, four key genes, namely *SOCS3*, *JUN*, *CLEC4C*, and *NFKBIA,* were identified by a protein–protein interaction (PPI) network and three machine learning algorithms. The results of functional enrichment revealed that *SOCS3*, *JUN*, and *NFKBIA* were all associated with hallmark TNF-α signaling via NF-κB. In addition, cells in JIA samples were clustered into four groups (B cell, monocyte, NK cell, and T cell groups) by single-cell data analysis. *CLEC4C* and *JUN* exhibited the highest level of expression in B cells; *NFKBIA* and *SOCS3* exhibited the highest level of expression in monocytes. Finally, real-time quantitative PCR (RT-qPCR) revealed that the expression of three key genes was consistent with that determined by differential analysis. Our study revealed four key genes with prognostic value for JIA. Our findings could have potential implications for JIA treatment and investigation.

## 1. Introduction

Juvenile idiopathic arthritis (JIA) is a general term for unexplained arthritis that persists for over six weeks in children younger than the age of 16 years; it is the most common chronic inflammatory rheumatic disease of childhood [1]. The global prevalence of JIA ranges from 15 to 400 cases per 100,000 individuals [2]. The International League Against Rheumatism has classified JIA into seven different categories based on the signs and symptoms that manifest within the first six months of onset. Despite receiving treatment, a few children respond unsatisfactorily to treatment, which can result in joint damage, irreversible disability, or severe extra-articular complications if not appropriately managed [3]. Although environmental factors and genetic susceptibility together are known to disrupt the balance between regulatory and effector immune cells, the pathophysiological mechanisms of JIA are yet to be fully elucidated [1]. The heterogeneity of JIA poses a challenge in predicting disease prognosis and outcome, underscoring the need for novel biomarkers and personalized treatment approaches.

The role played by the immune system in JIA as an autoimmune inflammatory disease is a crucial aspect that cannot be ignored. Various factors of adaptive immunity are known to most likely induce and maintain the persistence and long-term nature of JIA [1]. The complexity of the immune system is reflected in the diversity and plasticity of immune cell subpopulations and their functional phenotypes in JIA, indicating that immune cell heterogeneity may directly influence the development of JIA and its prognosis [4]. Therefore, prioritizing the investigation of immune cell-related differentially expressed genes (DEGs) and their influencing factors could potentially enhance our understanding of the underlying mechanisms of JIA.

Single-cell RNA sequencing (scRNA-seq) technology enables high-throughput and high-resolution analysis of precise gene expression patterns in individual cells, thereby providing a powerful approach to eliminating heterogeneity [5]. The workflow of scRNA-seq involves several steps, including single-cell acquisition, mRNA reverse transcription, cDNA amplification, cDNA library preparation, high-throughput sequencing, and data analysis [6]. The integration of single-cell multi-omics technologies with genomic, transcriptomic, proteomic, metabolomic, epigenomic, and spatial localization has enabled comprehensive analyses of biological systems, thereby creating novel avenues for investigating the heterogeneity of various physiologies and diseases. Recent developments in single-cell sequencing have been observed for a variety of autoimmune and inflammatory diseases, such as rheumatoid arthritis and systemic lupus erythematosus [7]. However, despite its potential, the application of scRNA-seq in JIA remains limited.

In this study, we performed a comprehensive analysis of transcriptome and scRNA-seq data to identify DEGs that might induce alterations in immune responses. This was achieved by examining homogeneous immune cell populations in both healthy individuals and patients with JIA. Subsequently, we predicted pathways and factors with regulatory potential, thereby exploring potential targets for prognostic and therapeutic interventions in JIA and elucidating potential immune modulation-related precision therapeutic strategies.

## 2. Results

### 2.1. Immune Cells Are Significantly Different between NC and JIA Samples

**Figure 1:**
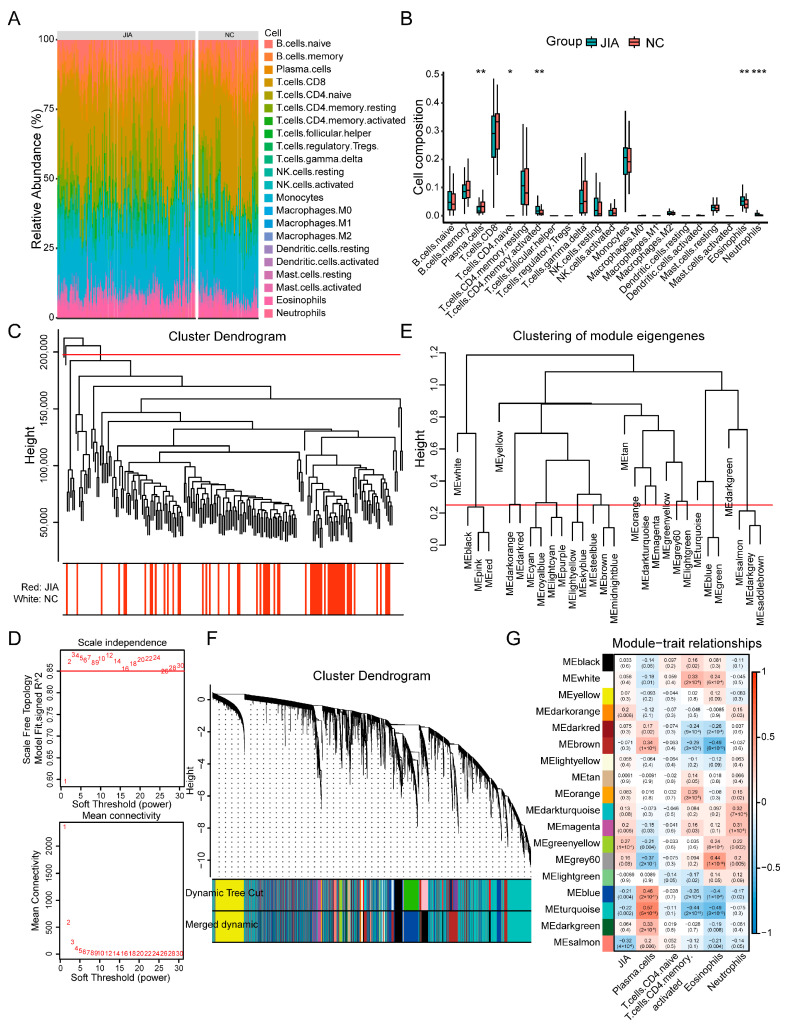
PBMC immune microenvironment and WGCNA of JIA and NC samples. (**A**) Abundance histogram of the proportion of immune cells. (**B**) A box plot of comparison of immune cells between JIA and NC samples. (**C**) Sample clustering dendrogram. (**D**) Network topology analysis under various soft-threshold and various soft-thresholding powers. The red line represents signed R^2^. (**E**) A dendrogram of module eigengenes. The red line represents MEDissThres value. (**F**) A gene dendrogram with different similarities and module colors. (**G**) A heat map of the correlations between 18 modules and JIA-related immune cells. *p* values are shown as * *p* < 0.05; ** *p* < 0.01; *** *p* < 0.001.

The progression of JIA is associated with an imbalanced immune microenvironment. Thus, the proportion of 22 immune cells in the samples in the training set was calculated using the CIBERSORT algorithm, and the results were visualized as an abundance histogram (Figure 1A). Then, we compared the proportion of immune cells in the NC and JIA samples. The results revealed that five types of immune cells, namely plasma cells, naive CD4 T cells, memory-activated CD4 T cells, eosinophils, and neutrophils, were significantly different in the JIA samples compared with those in the NC samples (Figure 1B and Figure S1). These immune cells were considered JIA-related immune cells.

### 2.2. A Total of 13,706 Genes Are Associated with JIA-Related Immune Cells

To identify genes associated with JIA-related immune cells, we determined genes exhibiting an expression level higher than one for clustering analysis. Two outlier samples, namely GSM340377 and GSM340368, were excluded (Figure 1C). Next, the value of the soft threshold (β) was determined to be two to validate that the reciprocity between genes was consistent with a scale-free distribution (Figure 1D). Then, 30 modules, each containing 30 genes, were obtained using a dynamic tree-cutting algorithm (Figure 1E). These modules were then merged (MEDissThres = 0.25) into 18 modules (Figure 1F). Finally, by performing correlation analysis, we identified four modules that showed high correlation (|R| > 0.4) with JIA-related immune cells. These modules were MEblue, MEbrowm, MEgray60, and MEturquoise, comprising a total of 13,706 genes (Figure 1G).

### 2.3. Differential Analysis Revealed 168 DE-ICRGs

**Figure 2:**
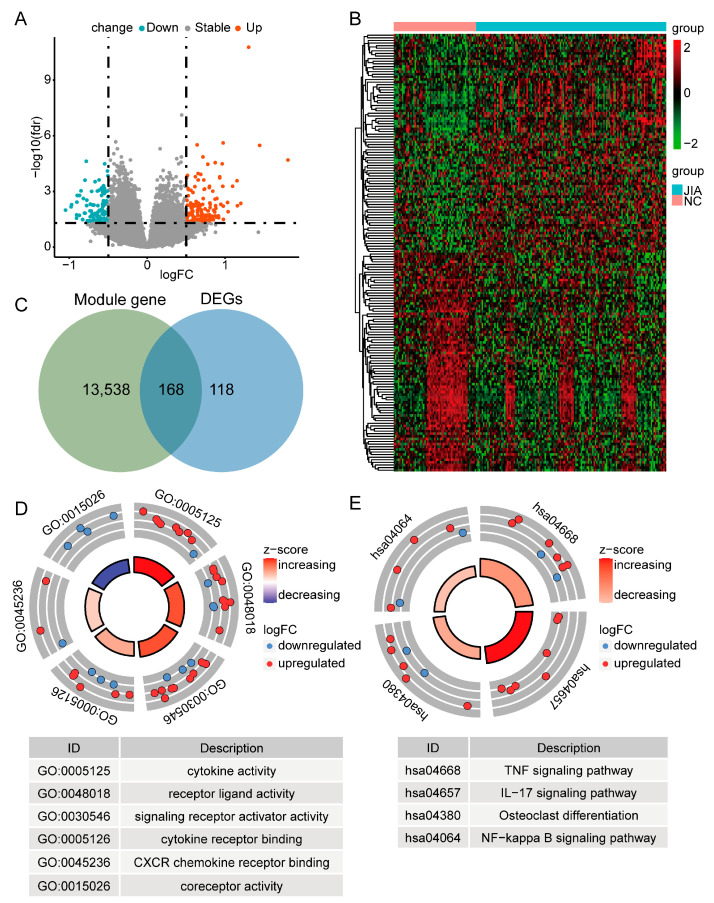
GO and KEGG enrichment analyses of DE-ICRGs in JIA. (**A**) A volcano plot of the DEGs. (**B**) A heat map of the DEGs. (**C**) A Venn diagram of genes identified by WGCNA and DEGs. (**D**) GO analysis of DE-ICRGs. (**E**) KEGG analysis of DE-ICRGs.

Differential analysis revealed 286 DEGs, including 168 upregulated genes and 118 downregulated genes, between JIA and NC samples (Figure 2A and Table S1). Figure 2B shows a heat map illustrating the expression level of each gene for each individual sample. Then, an intersection including 168 genes defined as DE-ICRGs was created by the Jveen website. The DE-ICRGs comprised 84 up- and 84 downregulated genes (Figure 2C). Additionally, enrichment analyses based on the expression of DE-ICRGs were performed. GO-MF analysis revealed six molecular functions, namely cytokine activity, receptor-ligand activity, signaling receptor activator activity, cytokine receptor binding, CXCR chemokine receptor binding, and coreceptor activity (Figure 2D and Figure S2). KEGG pathway enrichment analysis revealed four pathways, namely the TNF signaling pathway, IL-17 signaling pathway, osteoclast differentiation, and NF-κB signaling pathway (Figure 2E and Figure S3).

### 2.4. PPI Network and Machine Learning Analysis Revealed Key Prognostic Genes

**Figure 3:**
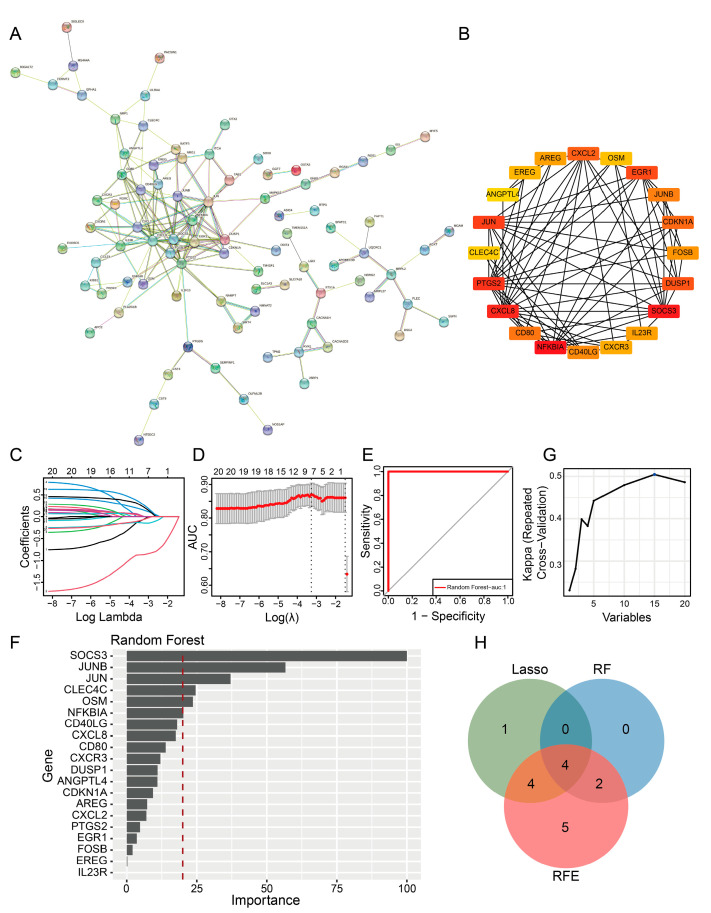
DE-ICRGs identified through PPI network and machine learning analysis. (**A**) PPI network of 168 shared genes, i.e., DE-ICRGs. (**B**) The top 20 genes exhibiting the highest degree of interaction in the PPI network. (**C**) LASSO regression model with gene coefficients, in which lines with different colors represent different genes. (**D**) LASSO regression model with 10-fold cross-validation. (**E**) ROC curves of the random forest model. (**F**) Feature importance scores of the random forest model. (**G**) Kappa of the RFE model. (**H**) Venn diagram of genes identified using the LASSO, random forest, and RFE models.

**Figure 4:**
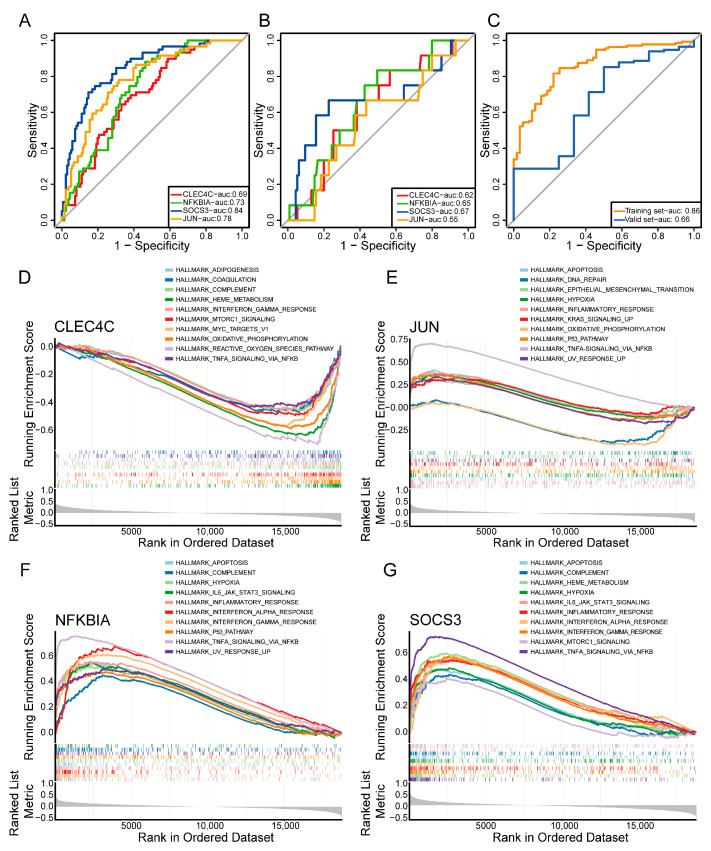
Validation of the hub genes and single-gene GSEA. (**A**) ROC curves of hub genes identified from the data sourced from GSE13501. (**B**) ROC curves of hub genes in identified from the data sourced from GSE112057. (**C**) ROC curves of logistic regression models for the key genes. (**D**–**G**) GSEA of *CLEC4C*, *JUN*, *NFKBIA*, and *SOCS3*. GSEA, gene set enrichment analysis; ROC, receiver operating characteristic.

After eliminating outlier proteins, we generated a PPI network based on the DE-ICRGs using the STRING database. The PPI network consisted of 156 nodes and 170 edges (Figure 3A). Then, using cytoHubba, we identified 20 genes, namely *NFKBIA*, *SOCS3*, *CXCL8*, *PTGS2*, *JUN*, *EGR1*, *DUSP1*, *CXCL2*, *CDKN1A*, *CD80*, *JUNB*, *CD40LG*, *FOSB*, *IL23R*, *AREG*, *CXCR3*, *EREG*, *OSM*, *CLEC4C*, and *ANGPTL4* (Figure 3B and Figure S4). Subsequently, three machine learning algorithms (LASSO, random forest, and RFE) were employed to further filter the genes that were identified. According to LASSO algorithm analysis, the best model fit was obtained when lambda was 0.00024262, and a total of nine genes were identified, namely *NFKBIA*, *SOCS3*, *JUN*, *CD80*, *CD40LG*, *IL23R*, *CXCR3*, *CLEC4C*, *and ANGPTL4* (Figure 3C,D). The area under the curve (AUC) of the ROC curve of the random forest model was equal to one, indicating that the model exhibited excellent performance in predicting JIA prognosis (Figure 3E). Subsequently, after the importance score was turned into a percentile, six genes (*SOCS3*, *JUNB*, *JUN*, *CLEC4C*, *OSM*, and *NFKBIA*) with scores greater than 20 were screened out by constructing the random forest model (Figure 3F). As shown in Figure 3G, kappa reached the maximum value when the number of features was 15, so 15 genes (*SOCS3*, *JUNB*, *JUN*, *OSM*, *CXCL8*, *NFKBIA*, *CLEC4C*, *CD40LG*, *CXCR3*, *ANGPTL4*, *CD80*, *CDKN1A*, *AREG*, *DUSP1*, and *EGR1*) were identified by the RFE algorithm. Then, these filtered genes were intersected. A total of four key genes with prognostic value in JIA, namely *SOCS3*, *JUN*, *CLEC4C*, and *NFKBIA*, were identified (Figure 3H). ROC curves generated based on the training and validation sets showed that the AUC of all key genes was greater than 0.5 (Figure 4A,B). Moreover, the key genes were used to construct a logistic regression model, and the ROC curve of the model was 0.86, indicating an excellent performance in predicting JIA prognosis (Figure 4C). The AUC value of the model was 0.66 in the validation set, which indicated the predictive power was decent (Figure 4C). Subsequently, to further explore the clinical diagnostic value of four key genes, we constructed a nomogram (C-index = 0.8605) to predict the risk of JIA (Figure S5A). The calibration curve and ROC curve (AUC = 0.86) demonstrated that the predictive power of the nomogram was excellent (Supplementary Figure S5B,C). Finally, we conducted single-gene GSEA to identify the top 10 pathways that showed a strong correlation with the key genes.

As shown in Figure 4D–G and Figures S6–S9, *CLEC4C* was enriched in pathways such as hallmark heme metabolism, hallmark oxidative phosphorylation, and hallmark myc targets v1 pathways; *JUN* was enriched in pathways such as hallmark TNF-α signaling via NF-κB, the hallmark inflammatory response, and hallmark apoptosis; *NFKBIA* was enriched in pathways such as hallmark TNF-α signaling via NF-κB, the hallmark interferon gamma response, and the hallmark interferon alpha response; and *SOCS3* was enriched in pathways such as hallmark TNF-α signaling via NF-κB, hallmark heme metabolism, and the hallmark interferon gamma response. The detailed results are provided in Tables S2–S5.

### 2.5. ScRNA-Seq Analysis of NC and JIA Samples

**Figure 5:**
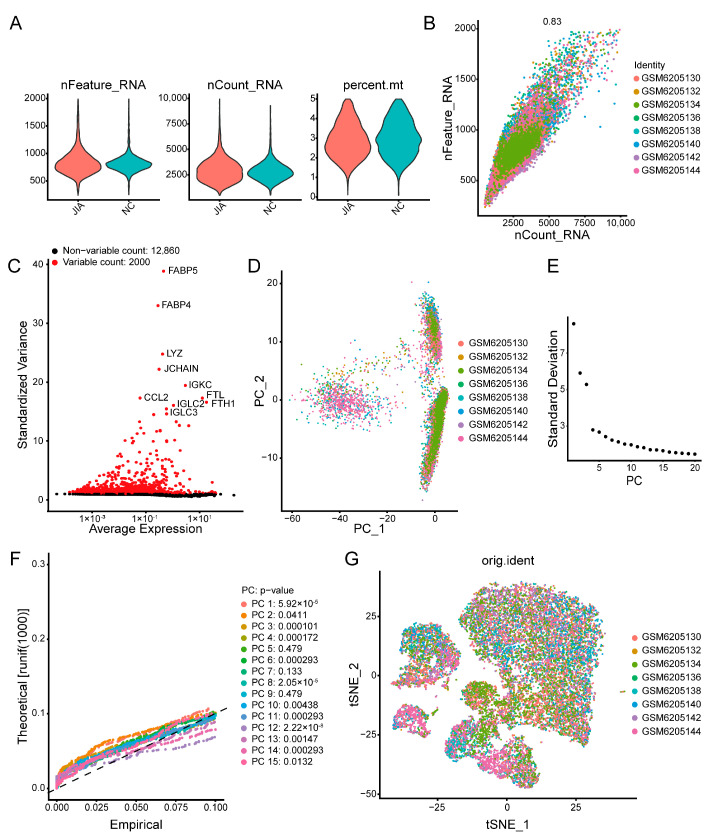
ScRNA-seq analysis of JIA and NC samples. (**A**) Violin plots following quality control and normalization. (**B**) Distribution of cell counts and intracellular gene counts following standardization. (**C**) A scatter plot of 2000 highly mutative genes and the top 10 among them. (**D**) PCA without distinct separations of cells in samples. (**E**,**F**) Elbow and JackStraw plots of PCs after linear dimensionality reduction. (**G**) A t-SNE plot of cell distribution in eight samples following t-SNE dimensionality reduction.

We downloaded scRNA-seq data pertaining to NC and JIA samples from a GEO dataset and identified cells with gene counts > 200 and <2000 and molecule counts < 10,000 (Figure 5A and Figure S10). Then, we conducted further filtering to identify cells with a percentage of mitochondrial genes below 5% (Figure 5B and Figure S11). Next, we screened 2000 highly mutative genes and identified the top 10 genes exhibiting the highest mutation rates, namely *FABP5*, *FABP4*, *LYZ*, *JCHAIN*, *IGKC*, *CCL2*, *FTL*, *IGLC2*, *FTH1*, and *IGLC3* (Figure 5C). No significant differences were observed in the overall distribution of the cells in each sample, indicating that the data were suitable for further analyses (Figure 5D). Linear dimensionality reduction processing was performed using the “JackStraw” and “ScoreJackStraw” functions, and 12 principal components were selected for further clustering of cells (Figure 5E,F). Then, nonlinear dimensionality reduction processing was performed using the “RunTSNE” function. The results revealed that the distribution of cells in each sample was uniform (Figure 5G). 

**Figure 6:**
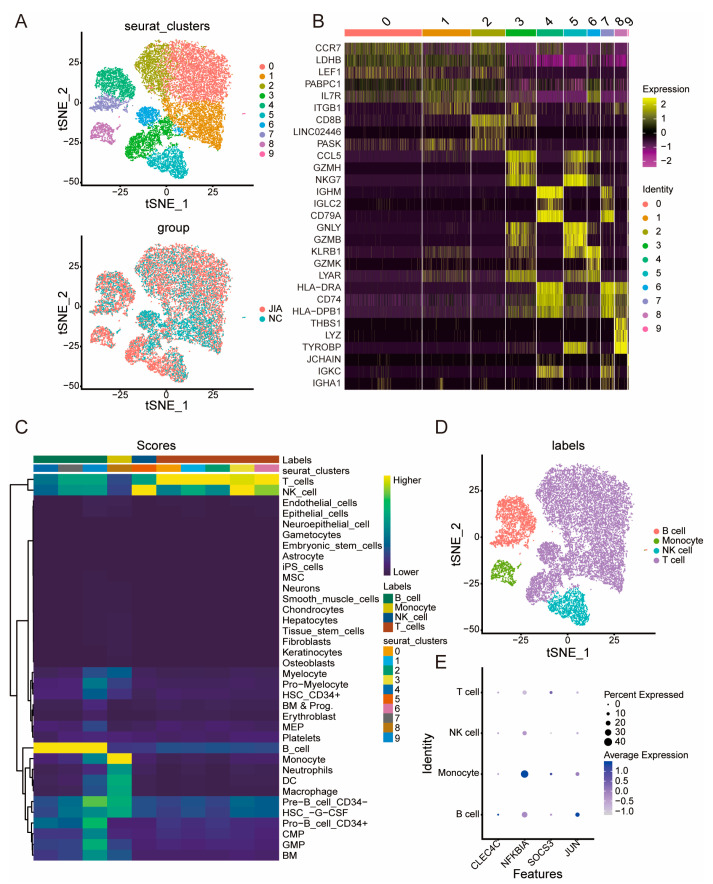
Clustering of immune cells and key gene expression patterns. (**A**) Clustering analysis and the constitution of clusters in JIA and NC. (**B**) A heat map of marker genes within nine clusters. (**C**) A heat map of cell annotation. (**D**) t-SNE visualization following cell subset annotation. (**E**) Dot plot of key gene expression patterns.

Following clustering analysis, cells in the samples were divided into 10 clusters. We found that the constitution of clusters corresponding to the JIA samples was similar to that of the NC samples; however, we observed a few differences in the cell counts in a few clusters, such as cluster 1, cluster 4, cluster 6, and cluster 9 (Figure 6A). Figure 6B shows the heat maps displaying the expression levels of the marker genes in different clusters, and Figure 6C shows the cell counts in different cell labels following annotation. Following annotation, cells were divided into four groups, namely B cell, monocyte, NK cell, and T cell groups (Figure 6D). As shown in Figure 6E, *CLEC4C* exhibited the highest level of expression in B cells; *NFKBIA* exhibited the highest level of expression in monocytes; *SOCS3* exhibited the highest level of expression in monocytes; and the *JUN* exhibited the highest level of expression in B cells. 

**Figure 7:**
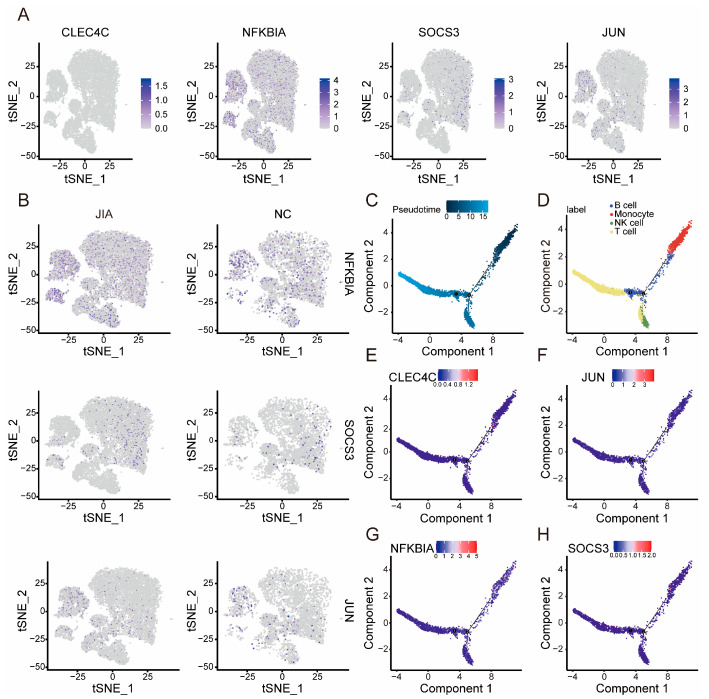
Cellular distribution of the key genes and cell quasi-temporal trajectory analysis. (**A**) Distribution of key genes in cells. (**B**) Comparison of the distribution of key genes in cells between JIA and NC samples. (**C**,**D**) Pseudotime analysis of the overall temporal alterations of cell populations. (**E**–**H**) The expression of *CLEC4C*, *JUN*, *NFKBIA*, and *SOCS3* as revealed by pseudotime analysis.

We mapped the key genes with respect to the distribution of the cells and found that the expression level of *NFKBIA*, *SOCS3*, and *JUN* in JIA samples was significantly higher than that in NC samples, whereas *CLEC4C* was not significantly expressed in the cells (Figure 7A,B). Next, 63 DEGs were identified among the clusters and used for the construction of quasi-temporal cell trajectories. Subsequently, we found that the cell population underwent temporal evolution, gradually transitioning from T cells to intermediate-type cells, such as B cells, before ultimately being replaced by monocytes or NK cells (Figure 7C,D). Finally, we observed the influence of key genes on the alterations in the cell populations and found that *JUN*, *NFKBIA*, and *SOCS3* exhibited correlations with alterations in the cell population (Figure 7E–H). 

### 2.6. Construction of the ceRNA Network of Key Genes

**Figure 8:**
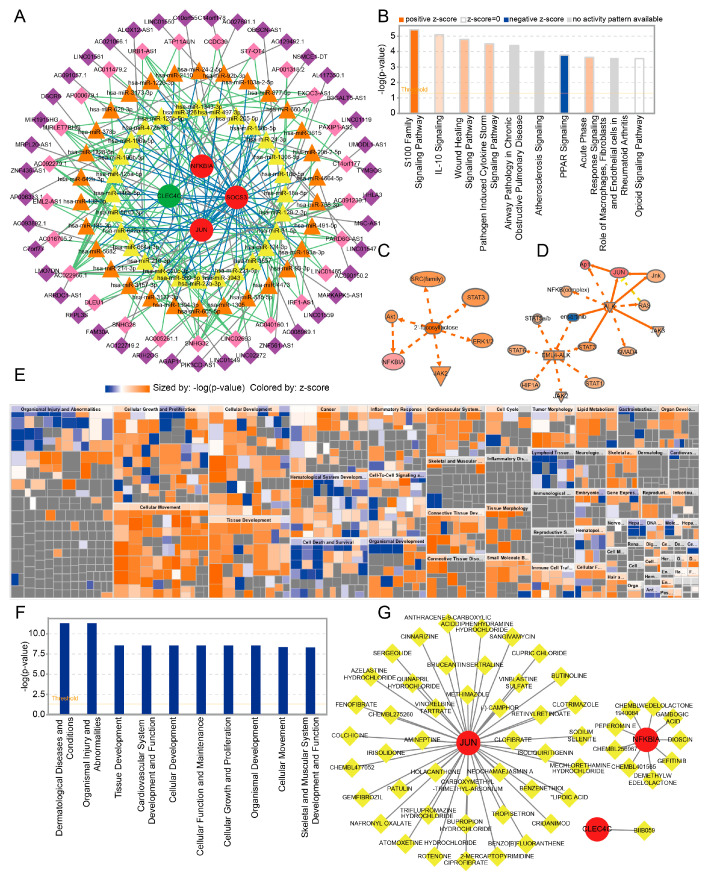
CeRNA network, IPA, and prediction of potential drugs targeting the key genes. (**A**) CeRNA network of key genes. Circles represent mRNA (red indicates upregulated expression, and green indicates downregulated expression); diamonds represent lncRNAs (purple indicates linkage = 1, and pink indicates linkage >1); and triangles represent miRNAs (orange indicates linkage = 2, and yellow indicates linkage >2). (**B**) The top 10 pathways as revealed by IPA. (**C**,**D**) The most significant upstream regulators of activation and inhibition and their pathways of action. (**E**,**F**) Identification of biological functions associated with the top 10 pathways as revealed by IPA. (**G**) The drug–gene interaction network.

We obtained 651 miRNAs that were associated with the key genes, and 788 mRNA–miRNA pairs were identified. Then, 66 lncRNAs associated with miRNA were obtained, and 145 lncRNA–miRNA pairs were identified. Finally, a ceRNA network based on miRNA–lncRNA pairs and miRNA–mRNA pairs was established. As shown in the network, 25 lncRNAs, including URB1-AS1 and ATP11AUN, exhibited more than one pairing relationship, and 28 miRNAs, including hsa-miR-326 and hsa-miR-497-3p, exhibited more than two pairing relationships (Figure 8A). Notably, URB1-AS1 was observed to regulate *SOCS3* in combination with hsa-miR-1343-3p, and MIRLET7BHG was observed to regulate *JUN* in combination with hsa-miR-125a-5p.

### 2.7. IPA and Prediction of Potential Drugs Targeting the Key Genes

DEGs between NC and JIA samples identified previously were observed to be enriched in 417 pathways. Figure 8B shows the top 10 pathways, including the S100 family signaling pathway, IL-10 signaling pathway, and wound healing signaling pathway. Then, we identified the most significant upstream regulator of activation, i.e., 2′-fucosyllactose 3, and the most conspicuous upstream regulator of inhibition, i.e., ensartinib 1 (Figure 8C,D). Then, we identified 70 biological functions, including dermatological diseases and conditions, organismal injury, and abnormalities (Figure 8E,F). Finally, we identified 44 potential drugs or molecular compounds with respect to *JUN*, such as colchicine and ciprofibrate. With respect to *NFKBIA*, we identified nine potential drugs or molecular compounds, including CHEMBL401565 and CHEMBL256967. With respect to *CLEC4C*, we identified only one function, i.e., BIIB059 (Figure 8G). The detailed results are provided in Tables S6–S8.

### 2.8. Validation of Key Genes by RT-qPCR

RT-qPCR revealed that the expression of three key genes, namely *SOCS3*, *JUN*, and *NFKBIA*, was significantly different between the MG and CG. These results were consistent with those of differential analysis (Figure S12).

## 3. Discussion

JIA is a common autoimmune inflammatory disease with a high disability rate in children and can involve multiple systems of the body [8]. As a heterogeneous disease, the immune system plays a crucial role in its pathogenesis; however, its mechanism remains unclear [1]. Given the challenges in meeting the clinical requirements through conventional treatments and the potential for the persistence of the disease or permanent damage [9], predictive biomarkers and rapid and precise therapeutic alternatives are necessary. ScRNA-seq has powerful applications in immunology owing to its ability to reveal cellular composition, transcriptional dynamics, and networks of gene regulatory relationships through unbiased high-throughput and high-resolution analysis of individual cells, thereby adding an additional dimension to the field [10]. The utilization of scRNA-seq technology and associated data analysis methods may facilitate the elucidation of the mechanisms underlying immune cell heterogeneity in JIA.

Regarding the immunopathogenesis of JIA, adaptive immunity may be responsible for the onset and persistence of the disease [1], whereas the innate immune-driven inflammatory response plays a key role in mediating autoimmune damage [4]. Thus, genes that affect either inflammatory or immune processes could be potential targets for combating JIA. In this study, four ICRGs were identified to possess prognostic value for JIA. 

*CLEC4C*, a transmembrane protein and a member of the C-type lectin superfamily, serves as the most specific protein marker for plasmacytoid dendritic cells (pDCs) [11]. The activation and remodeling of the immune system have been elucidated to be primarily facilitated by C-type lectin receptors [12]. Antibodies that are conjugated to *CLEC4C* are efficiently loaded onto MHC-II for presentation to T cells following internalization by pDCs; activated pDCs then release IFN-I and pro-inflammatory cytokines [13], which may contribute to the development of autoimmune diseases, such as rheumatoid arthritis. *CLEC4C* was also presented as a risk factor for short-term clinical relapse of Crohn’s disease [14]. Cross-linking *CLEC4C* using specific monoclonal antibodies can inhibit IFN-I secretion by pDCs [13]. Consequently, mitigating autoimmune diseases resulting from exaggerated IFN-I responses may be possible. Nonetheless, the role of IFN-I in JIA is yet to be elucidated.

*AP-1*, a homo- or hetero-dimeric transcriptional complex that is primarily composed of the members of the *Jun* (*c-Jun*, etc.) and FOS protein families [15], is implicated in a variety of biological processes and disease mechanisms. The activation of the AP-1 signaling pathway can be induced by LPS and TNF-α, which results in the increased expression of inflammatory cytokines [16]. JUN binding to FOS plays a role in various steps of establishment, maintenance, and recall of inflammatory memory [17]. In addition, *AP-1* affects several aspects of the immune response mechanism [15]. For example, *c-Jun* is a key positive regulator of CCL2 and IL-23 expression in DC and is therefore closely associated with chronic autoimmune diseases, such as psoriasis [18]. The lipopolysaccharide-induced inflammatory response could be suppressed by promoting the ubiquitination and degradation of c-Jun [19]. The suppression of AP-1 exacerbated STING-induced immune responses as well [20]. Targeting *AP-1* and *JUN* could be one of the potential therapeutic approaches against inflammatory and immune-related diseases, including JIA.

The *NFKBIA* gene, located on chromosome 14q13, is composed of six exons spanning 3.5 kbp and encodes the most common protein of the *IκB* family, i.e., IκBa. IκBa is sequestered in a complex inactive state with NF-κB in the cytoplasm. Upon stimulation, the complex is rapidly degraded, which results in the release and translocation of NF-κB to the nucleus, wherein it initiates inflammation-associated gene transcription [21]. Therefore, IκBa serves as an *NF-κB* repressor protein. The activation of *NF-κB* plays a crucial role in both innate and adaptive immunity. The first meta-analysis aimed at investigating the relationship of *NFKBIA* gene polymorphisms with autoimmune and inflammatory diseases demonstrated that *NFKBIA* gene promoter -826C/T polymorphism increased the risk and might have a general effect on predisposition to these diseases [22]. Another study showed that a novel *NFKBIA* variant caused immunodeficiency, manifested in JIA [23]. Additionally, the *NFKBIA* gene has been found to exhibit polymorphisms that could potentially serve as viable candidates for investigating susceptibility to auto-inflammatory immune diseases, such as JIA [24].

*SOCS3* is a key physiological regulator of STAT3 signaling and has complex functions in both innate and adaptive immunity. *SOCS3* functions as a negative regulator of the JAK/STAT3 signaling pathway by binding to JAK and cytokine receptors, leading to the inhibition of STAT3 phosphorylation, ultimately resulting in a protective immune response against infectious and inflammatory diseases [25]. The inhibition of SOCS3 ubiquitination and proteasomal degradation led to protein accumulation and stronger inhibition of IL-6 signaling and barrier function loss [26]. The Th17 cell subpopulation is known to be crucial in the development and pathogenesis of autoimmune diseases; STAT3 activation results in the expression of the RORγt transcription factor and the differentiation of Th17 cells [27]. STAT3 phosphorylation is mediated by IL-23, highlighting the potential regulatory role of *SOCS3* and *JUN* in JIA, and their expression is consistent with the results of our study. pDC is a rare population of leukocytes in the peripheral blood, accounting for less than 0.4% of PBMCs [11], whereas IFN-I-producing cells are enriched in synovial fluid in JIA [28]. Therefore, *CLEC4C* expression may not be evident in PBMCs. However, all four genes regulate different mechanisms in inflammatory and immune processes, indicating their potential relevance to JIA.

IPA revealed 10 pathways significantly associated with the four key genes. Among them, the S100 protein family, the most abundant alarm protein in juvenile rheumatic diseases, is released as an endogenous protein during stress response and transmits inflammatory signals through pattern recognition receptors; therefore, its expression and stability can serve as a biomarker for monitoring JIA [29]. IL-10, a well-known immunosuppressive and anti-inflammatory cytokine, can impede the development of collagenous arthritis by preserving the function of Treg cells [30]. The cytokine storm is a series of clinical manifestations due to excessive activation of the immune system and is commonly observed in auto-inflammatory diseases; the IL-10 signaling pathway mediates the pathogen-induced production of pro-inflammatory factors in immune cells [31]. Macrophages, fibroblasts, and endothelial cells are involved in the pathological processes associated with rheumatoid arthritis [32], and they may exhibit alterations in the pathological processes of JIA. Therefore, the key genes may be involved in the pathological processes of JIA through enriched signaling pathways and could serve as critical target pathways for regulation.

Among the upstream regulators of the DEGs, ensartinib exhibited the most significant inhibitory effect. It is a novel ALK inhibitor that has been approved for advanced ALK-positive non-small cell lung cancer indications following treatment with crizotinib [33]. Lipolytic products of triglyceride-rich lipoproteins have been elucidated to inhibit the expression of pro-inflammatory genes associated with AP-1 signaling and NF-κB signaling via ALK inhibition [34]. However, whether ensartinib can affect JIA by regulating associated genes remains unclear. The most significantly activated upstream regulator was 2′-fucosyllactose. It is an oligosaccharide found abundantly in breast milk and has many important biological properties, including antibacterial, antiviral, and immunomodulatory properties [35]. It may exert anti-inflammatory effects by targeting the TLR4/MyD88/NF-κb-related inflammatory pathway through the upregulation of NLRP6 expression [36]. 2′-fucosyllactose has also been elucidated to inhibit Th17 cell immune responses and the release of associated cytokines through the STAT3 signaling pathway [37]. Therefore, 2′-fucosyllactose may exhibit anti-inflammatory effects in JIA. Biological functions such as cellular and tissue development, cellular function and maintenance, cellular growth and proliferation, and skeletal and muscular system development and function are closely associated with JIA. Therefore, the DEGs associated with JIA may play a role in JIA by affecting these biological functions.

Among the 54 potential drug targets postulated to regulate the key genes in this study, potential targets associated with the *JUN* gene included various drugs that have been approved for use, such as antihistamines, lipid regulators, antibiotics, antitumor agents, neurological agents, and others. Among these, diphenhydramine hydrochloride, an antihistamine has been employed to inhibit Th17-related autoimmune inflammatory diseases [38]; tropisetron, a 5-HT3 receptor antagonist, has been shown to balance the immune response through the JAK2/STAT3 signaling pathway [39]; lipid-regulating fibrates, which are well-recognized anti-inflammatory agents, such as fenofibrate, have been shown to both inhibit Th17 cells [40] and enhance the differentiation of mouse Foxp3+ Tregs cells in vitro [41]; clofibrate has been shown to reduce inflammatory signaling by suppressing NF-κB translocation [42]; clotrimazole, an antifungal agent, has been shown to enhance the T-cell response by modulating dendritic cell-mediated antigen presentation [43]; and colchicine, which is employed for the clinical treatment of gouty arthritis, has been elucidated to possess broad anti-inflammatory properties and reduce cytokine production by impeding the activation of inflammatory vesicles [44].

Among the potential drug targets associated with the *NFKBIA* gene, the bioactive compounds included wedelolactone, dioscin, peperomin E, and gambogic acid. The active ingredients wedelolactone, dioscin, and peperomin E have been shown to exhibit blocking effects on NLRP3 inflammatory vesicles [45,46,47]. Gambogic acid has been shown to induce anti-inflammatory effects in collagen-induced arthritis by reducing the concentrations of TNF-α, IL-6, and other cytokines [48]. These aforementioned active compounds exhibit potential utility in the treatment of arthritis or inflammation. BIIB059, a monoclonal antibody (anti-CLEC4C) developed for application in SLE, rapidly internalizes pDC surface receptors by binding to *CLEC4C*, thereby effectively inhibiting the production of IFN-1, cytokines, and chemokines [49]. The other compounds, such as isoliquiritigenin [50] and irisolidone [51], have not been utilized clinically. These compounds exhibit anti-inflammatory and modulating immune activity. Therefore, they possess clinical therapeutic potential and warrant further investigation. The new biologic disease-modifying anti-rheumatic drugs and small molecules approved for treating JIA were expensive [1]. These compounds may have a higher effect–cost ratio and provide more options for the treatment of JIA.

Given the heterogeneity of JIA subtypes and the need for evidence based on larger clinical datasets, the results obtained so far have limitations, and the precise elucidation of JIA remains challenging. Therefore, to prudently explain the complexity of JIA and develop therapies that hold potential for disease mitigation, obtaining and analyzing larger clinical samples while also conducting additional experimentation to validate the key genes, targets, and potential regulatory mechanisms that have been elucidated is imperative. Considering the samples regarding the JIA datasets used in the study were all from blood, we believe that the nomogram could be considered as an effective clinical diagnostic and practical device with the help of the peripheral blood tests and evaluation of the key gene expressions.

## 4. Materials and Methods

### 4.1. Data Acquisition

RNA sequencing data (RNA-seq) of peripheral blood mononuclear cell (PBMC) samples from normal controls (NC) and patients with JIA were downloaded from the Gene Expression Omnibus (GEO) cohort (http://portal.gdc.cancer.gov/ (accessed on 28 September 2022)). The GSE13501 dataset, which comprised 59 NC samples and 136 JIA samples, was used as the training set, and the GSE112057 dataset, which comprised 202 JIA samples, was used as the validation set. Additionally, the scRNA-seq data of JIA was sourced from the GSE205095 microarray, which comprised six JIA samples and two NC samples.

### 4.2. Immune Microenvironment Analysis of NC and JIA Samples

The proportion of 22 types of immune cells in the NC and JIA samples in the training set was calculated using the cell type identification by estimating relative subsets of the RNA transcripts (CIBERSORT) algorithm. The resulting data were visualized as an abundance histogram [52]. The proportions of immune cells in the NC and JIA samples (*p* < 0.05) were compared using the rank sum test. The results were presented as a box plot and a radar plot.

### 4.3. Identification of Immune Cell-Related Genes (ICRGs)

Weighted gene co-expression network analysis (WGCNA) was performed to identify ICRGs using the “WGCNA” package in R (version 1.71). Initially, clustering analysis was performed to eliminate outlier samples to ensure the accuracy of subsequent analyses. Next, the soft threshold (β) was determined to ensure that the reciprocity among genes conformed to scale-free distribution. Then, a dynamic tree-cutting algorithm was employed to cluster genes into distinct modules. Finally, correlation analysis was performed to determine correlations between modules in the network and JIA-related immune cells. The results were visualized as a heat map.

### 4.4. Differential Expression and Enrichment Analysis of ICRGs

The “limma” package in R (version 3.44.3) was used to identify DEGs between NC and JIA samples based on the training set, with a significance threshold of *p* < 0.05 and |log2FC| > 0.5 [53]. Then, an intersection between genes identified in the WGCNA and DEGs was generated using the jveen website (http://jvenn.toulouse.inra.fr/app/example.htm (accessed on 29 September 2022)). Intersecting genes were designated as differentially expressed ICRGs (DE-ICRGs). Thereafter, the “clusterProfiler” package in R (version 3.8.1) was used to conduct functional and pathway enrichment analyses of the DE-ICRGs (adj. *p* < 0.05) using the Gene Ontology (GO) and Kyoto Encyclopedia of Genes and Genomes (KEGG) databases [54].

### 4.5. Protein–Protein Interaction (PPI) Network and Machine Learning Analysis of DE-ICRGs

The STRING database (https://cn.string-db.org/ (accessed on 30 September 2022)) was used to generate a PPI network of the DE-ICRGs. Then, the molecular composite detection algorithm was employed to analyze the modules in the PPI network. Finally, the cytoHubba tool in the “Cytoscape” package in R (version 3.8.2) was used to identify the genes showing the greatest degree of interactions, which were then designated candidate genes [55]. Then, the least absolute shrinkage and selection operator (LASSO) algorithm of the “glmnet” package in R (version 4.1-1) was used to filter candidate genes [56]. Then, the “caret” package in R (version 6.0-92) was used to employ the random forest algorithm and construct a random forest model for filtering candidate genes. Next, the recursive feature elimination (RFE) algorithm in the “caret” package in R was used to filter candidate genes using the “rfe” function. Finally, the genes yielded by the three aforementioned machine learning algorithms were intersected to identify key genes with prognostic value for JIA.

On the basis of the training and validation sets, we verified the prognostic performance of the key genes by receiver operating characteristic (ROC) analysis. Then, a logistic regression model based on the key genes was developed and validated using the training and validation sets. Finally, single-gene gene set enrichment analysis (GSEA) was performed to enriched functions of key genes by using the “GSVA” package in R (version 1.36.3) based on hallmark gene sets from the MSigDB database (https://www.gsea-msigdb.org/gsea/msigdb/human (accessed on 10 October 2022)) [57].

### 4.6. ScRNA-Seq Analysis of NC and JIA Samples

To adjust and normalize scRNA-seq data, we used the “CreateSeuratObject” function in the “Seurat” package in R (version 4.0.2). Specifically, the data were filtered based on the criteria of 200 < n.features < 2000 and n.Count < 10,000. The expression of mitochondria genes was determined using the “PercentageFeatureSet” function. Cells with a percentage of mitochondrial genes below 5% were further analyzed. Then, we integrated the “FindVariableFeatures” function and the “vst” tool to filter the top 2000 genes showing the highest mutation rates and subsequently generated scatter plots. 

Principal component analysis (PCA) was performed to validate the uniformity of the overall distribution of cells in the samples. Next, the “JackStraw” function and “ScoreJackStraw” function were used to conduct PCA repeatedly to calculate the *p*-value of genes in each principal component. Then, the top 12 principal components were selected for the clustering of cells in the samples. The “RunTSNE” function was used to execute the t-SNE dimensionality reduction process. Then, clustering analysis was performed by integrating the “FindNeighbors” and “FindClusters” functions, and the cells were divided into various clusters. Next, the “SingleR” package in R (version 1.6.1) was used to annotate the cells, and t-SNE visualization was conducted. Thereafter, DEGs were identified as feature genes among clusters based on a significance threshold of *p* < 0.01. The DDRTree method was employed to construct quasi-temporal cell trajectories. Finally, based on the trajectories, the alterations in the feature genes with respect to the alterations in the cell populations were determined.

### 4.7. Construction of a Competing Endogenous RNAs (ceRNA) Network

The “mirwalk” software (http://mirwalk.umm.uni-heidelberg.de (accessed on 13 October 2022)) was used to predict micro RNAs (miRNAs) associated with the key genes and determine mRNA–miRNA pairs. Then, long non-coding RNAs (lncRNAs) that were associated with the miRNAs were acquired from the starBase database (https://starbase.sysu.edu.cn/ (accessed on 13 October 2022)), and lncRNA–miRNA pairs were determined. Finally, a ceRNA network was constructed.

### 4.8. Ingenuity Pathway Analysis (IPA) and Prediction of Potential Drugs Targeting the Key Genes

IPA was performed using the QIAGEN IPA database (www.qiagen.com/ingenuity (accessed on 11 October 2022)) based on the expression of the identified DEGs between JIA and NC samples. IPA is composed of three distinct components, namely classical pathway analysis, upstream regulators analysis, and biological functional analysis. Finally, potential drugs or molecular compounds targeting the key genes were identified using the Drug-Gene Interaction Database (https://dgidb.genome.wustl.edu/ (accessed on 11 October 2022)).

### 4.9. In Vivo Experiments

Following one week of acclimatization feeding, SPF DBA/1 mice (male, six-week-old, 18-20 g) were randomly divided into two groups (*n* = 6) as follows: the model group (MG, type II bovine collagen + Freund’s complete adjuvant/Freund’s incomplete adjuvant) and NC group (CG, saline). On day 0, the mice in the MG were subcutaneously injected with 100 μL of 1:1 type II bovine collagen and Freund’s complete adjuvant (Chondrex, Redmond, WA, USA) at multiple points of the caudal root. After 21 days, the same mice were injected with 1:1 type II bovine collagen + Freund’s incomplete adjuvant (Chondrex, Redmond, WA, USA). As per established scoring criteria [58], a score > 4 was considered to indicate successful modeling. Following successful modeling, peripheral blood was obtained from the mice, and PBMCs were isolated according to the instructions of the whole blood single nucleated cell isolation solution (Solarbio, Beijing, China). Total RNA was extracted by using TRIzol (Thermo Fisher, Waltham, MA, USA). This study was approved by the Guangzhou Medical University Ethics Committee.

### 4.10. Real-Time Quantitative PCR (RT-qPCR)

Six MG and six CG PBMC samples were fully lysed and centrifuged to obtain the upper aqueous phase. Next, isopropanol was added, and a series of treatments were performed to obtain a clear RNA precipitate. Then, RNA was reverse-transcribed using the SureScript^TM^ First-strand cDNA Synthesis kit (Invitrogen, Carlsbad, CA, USA) to obtain cDNA for subsequent RT-qPCR assays. The relevant primer sequences are listed in Table 1.

### 4.11. Statistical Analysis

All statistical analyses were performed using R (version 4.2.0). The “ggplot2” package in R (version 3.3.0) was used to generate volcano plots and box plots [59]. The “pheatmap” package in R (version 1.0.12) was employed to generate heat maps. The “survivalROC” package in R (version 1.0.3) was employed to plot the ROC curves.

## 5. Conclusions

In this study, by analyzing transcriptome and scRNA-seq datasets using machine learning algorithms, we showed that five types of immune cells are different in JIA samples. We identified four key genes associated with immune cells in JIA and predicted the associated classical pathways, regulatory factors, and potential targets. Our study showed that these four genes play different key roles in inflammation and immune regulation, indicating that the differential expression of these genes may be crucial for specific treatment and predicting the disease prognosis in JIA. This study provides a basis for the comprehensive exploration of immune heterogeneity in JIA and the improvement of personalized therapeutic strategies against JIA.

## Figures and Tables

**Table 1:** Primer sequences.

Primers	Sequence
Nfkbia F	ATGGAAGTCATTGGTCAGGTGA
Nfkbia R	ACAGGCAAGATGTAGAGGGGTA
Socs3 F	CAGTCGGGGACCAAGAACCTA
Socs3 R	GTACACAGTCGAAGCGGGGAA
Jun F	ACGCCAACCTCAGCAACTT
Jun R	TCCTCATGCGCTTCCTCTC
Internal reference-M-Gapdh F	CCTTCCGTGTTCCTACCCC
Internal reference-M-Gapdh R	GCCCAAGATGCCCTTCAGT

## Data Availability

The datasets used during the current study are available from the corresponding author upon reasonable request.

## Supplementary Materials

The following supporting information can be downloaded at: https://www.mdpi.com/article/10.3390/ijms241310619/s1.
