# Supplementary material for: Immune Cell-Related Genes in Juvenile Idiopathic Arthritis Identified Using Transcriptomic and Single-Cell Sequencing Data
Source: Int J Mol Sci. 2023 Jun 25;24(13):10619. doi: 10.3390/ijms241310619 (PMC10342059; doi:10.3390/ijms241310619)
Supplement: Supplementary file 1 [file ijms-24-10619-s001.zip › Supplementary Files.pdf]

Supplementary Materials: Table S1. DEGs (n = 286) between JIA and NC samples sourced from the GSE13501 dataset; Table S2. Hallmark pathways associated with CLEC4C as revealed by GSEA; Table S3. Hallmark pathways associated with JUN as revealed by GSEA; Table S4. Hallmark pathways associated with NFKBIA as revealed by GSEA; Table S5. Hallmark pathways associated with SOCS3 as revealed by GSEA; Table S6. Pathways (n = 417) enriched by classical pathway analysis; Table S7. Diseases and biological functions (n = 70) associated with the key genes; Table S8. Upstream regulators of key genes; Figure S1. Radar map depicting the abundance of immune cells; Figure S2. Chord plot of GO enrichment analysis; Figure S3. Dot map of KEGG enrichment analysis; Figure S4. Topological attribute analysis diagram of DE-ICRGs, in which different color symbols represent different DE-ICGs; Figure S5. Construction and validation of the nomogram. (A) The nomogram (C-index = 0.8605) established based on four key genes. (B) The calibration curve of the nomogram. (C) The receiver operating characteristic (ROC) curve of the nomogram. AUC, area under the curve; Figure S6. Dot plot of gene set enrichment analysis of CLEC4C; Figure S7. Dot plot of gene set enrichment analysis of JUN; Figure S8. Dot plot of gene set enrichment analysis of NFKBIA; Figure S9. Dot plot of gene set enrichment analysis of SOCS3; Figure S10. Violin diagram before quality control and normalization; Figure S11. Distribution of cell counts and intracellular gene counts before standardization; Figure S12. Real-time quantitative PCR of the three key genes. * $p < 0.05$; *** $p < 0.001$.
