# Supplementary figures and images for: Immune Cell-Related Genes in Juvenile Idiopathic Arthritis Identified Using Transcriptomic and Single-Cell Sequencing Data
Source: Int J Mol Sci. 2023 Jun 25;24(13):10619. doi: 10.3390/ijms241310619 (PMC10342059; doi:10.3390/ijms241310619)

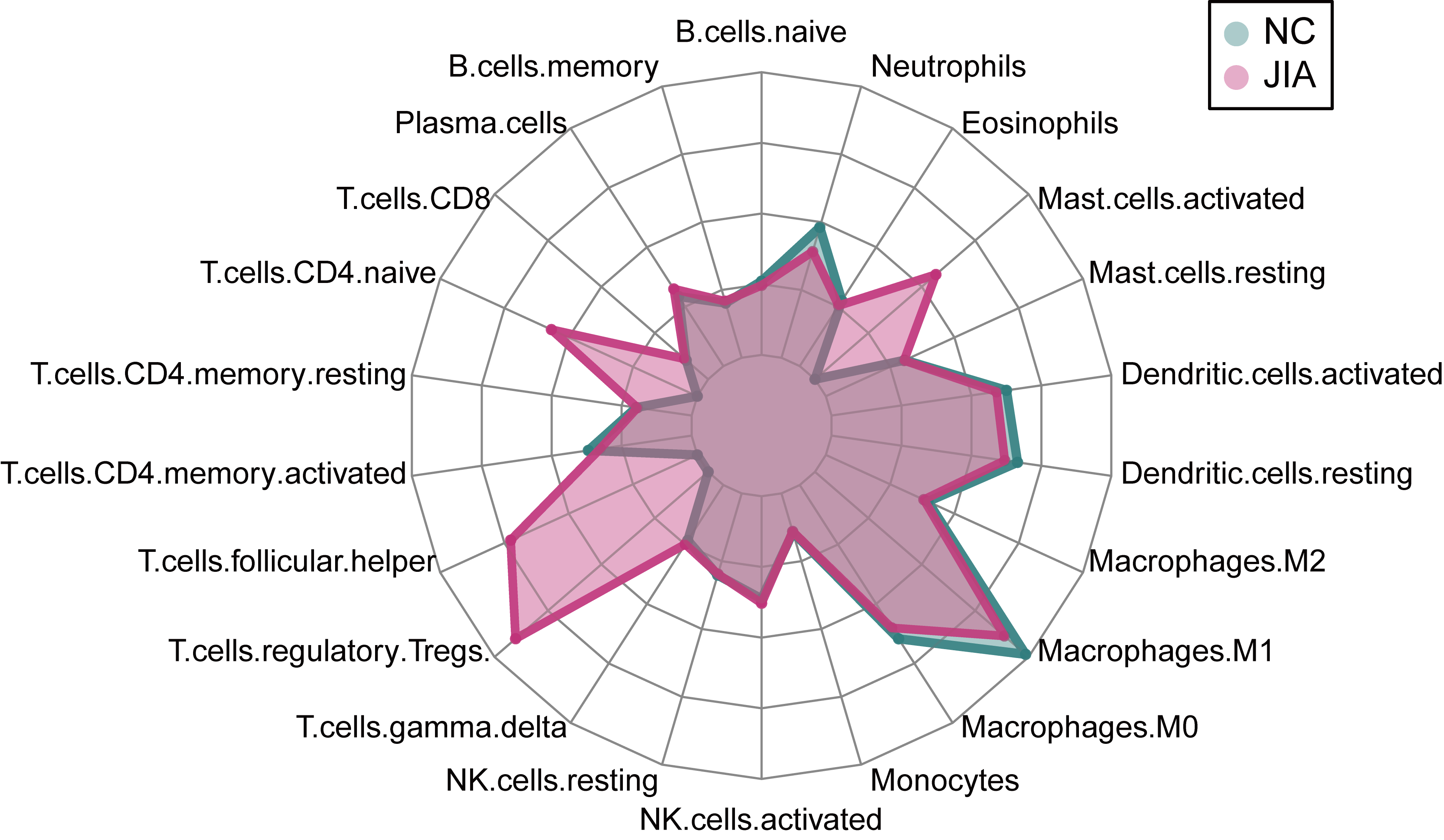

Supplement: Supplementary file 1 [file ijms-24-10619-s001.zip › Supplementary figures/Figure S1.tif]

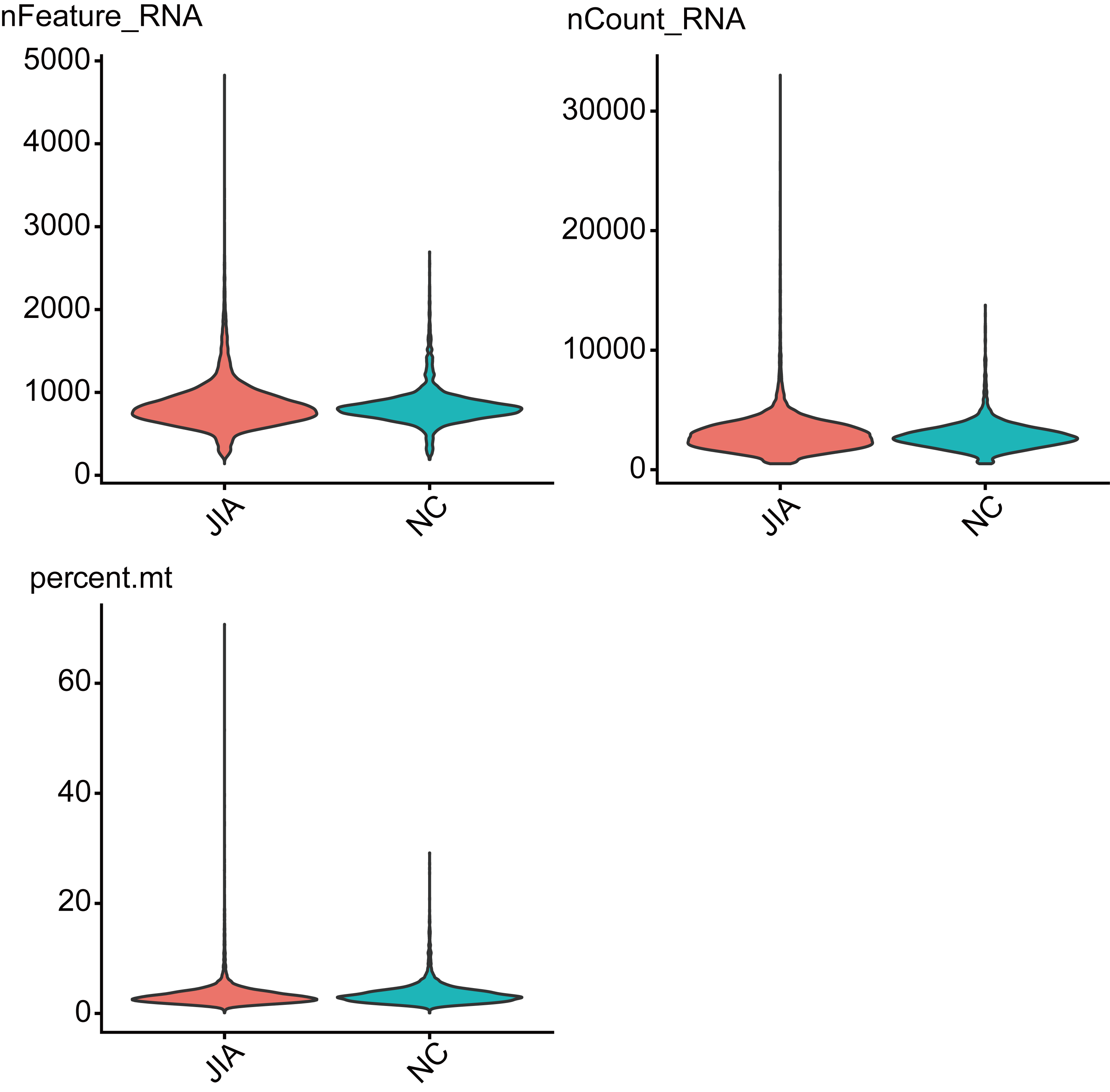

Supplement: Supplementary file 1 [file ijms-24-10619-s001.zip › Supplementary figures/Figure S10.tif]

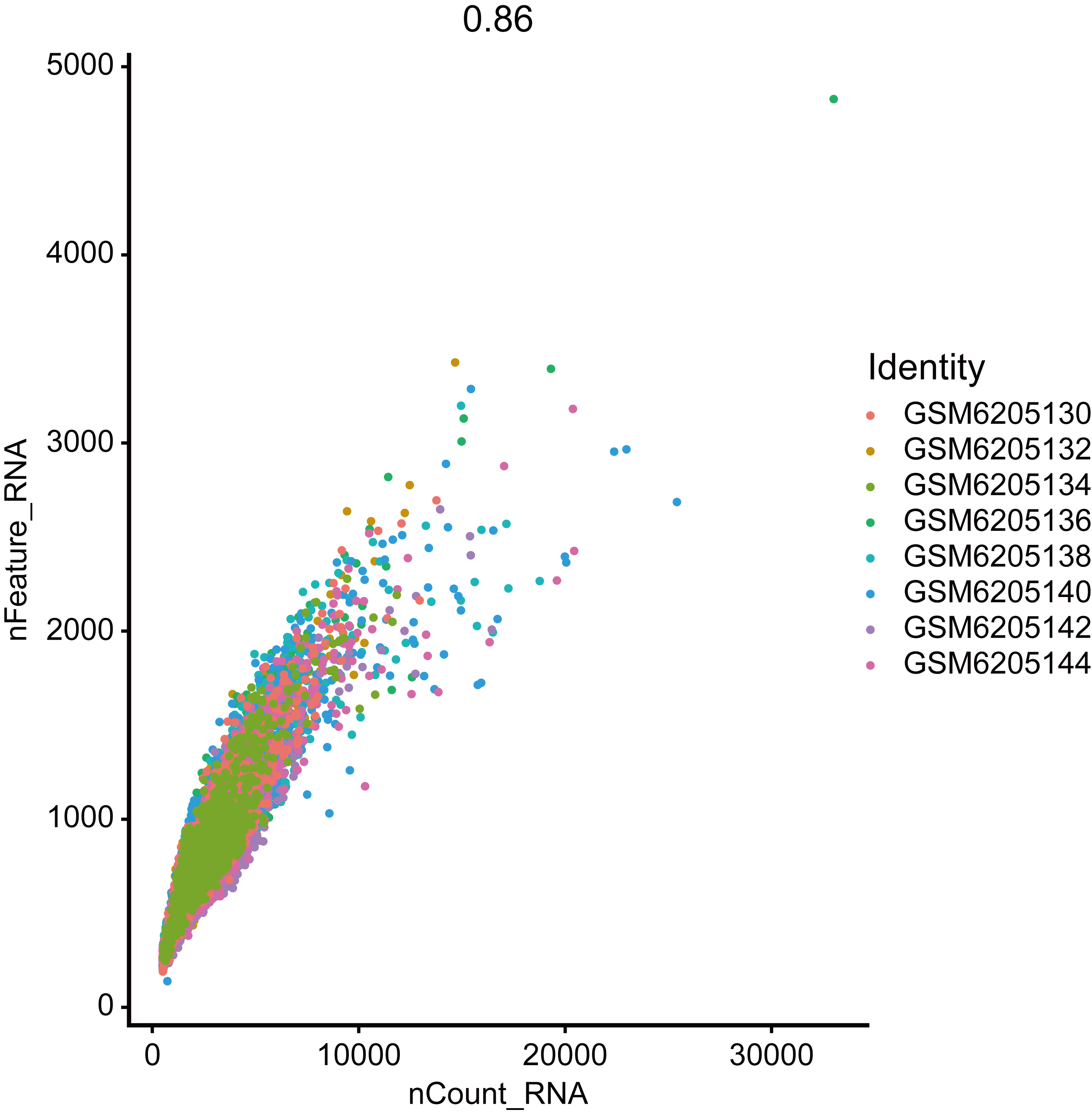

Supplement: Supplementary file 1 [file ijms-24-10619-s001.zip › Supplementary figures/Figure S11.tif]

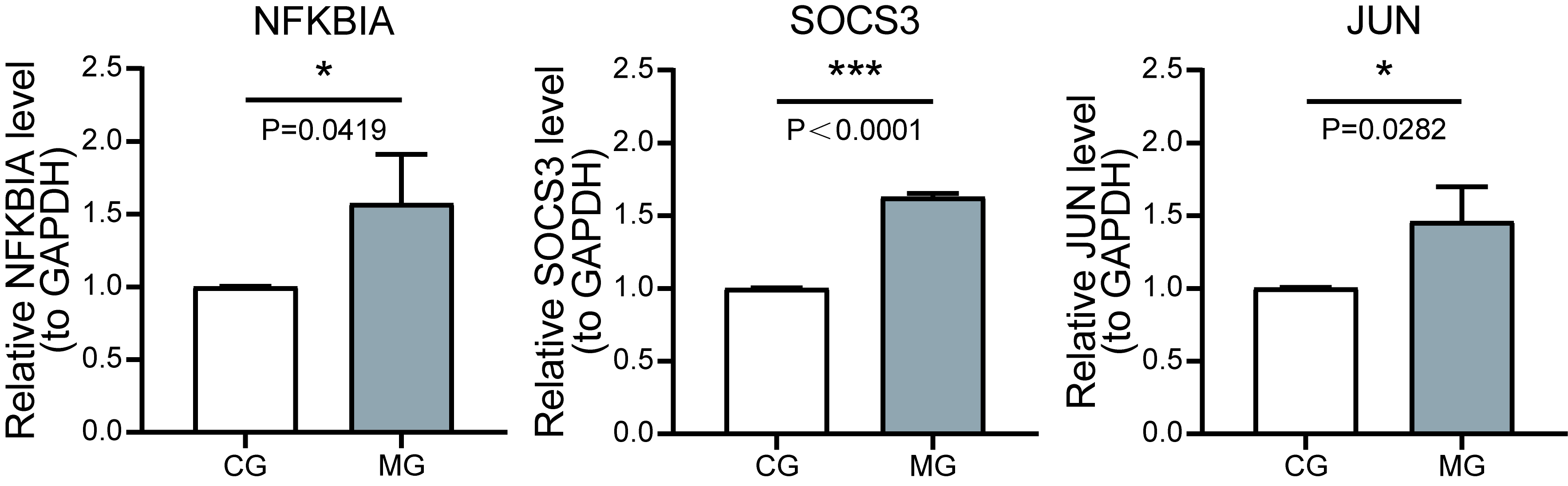

Supplement: Supplementary file 1 [file ijms-24-10619-s001.zip › Supplementary figures/Figure S12.tif]

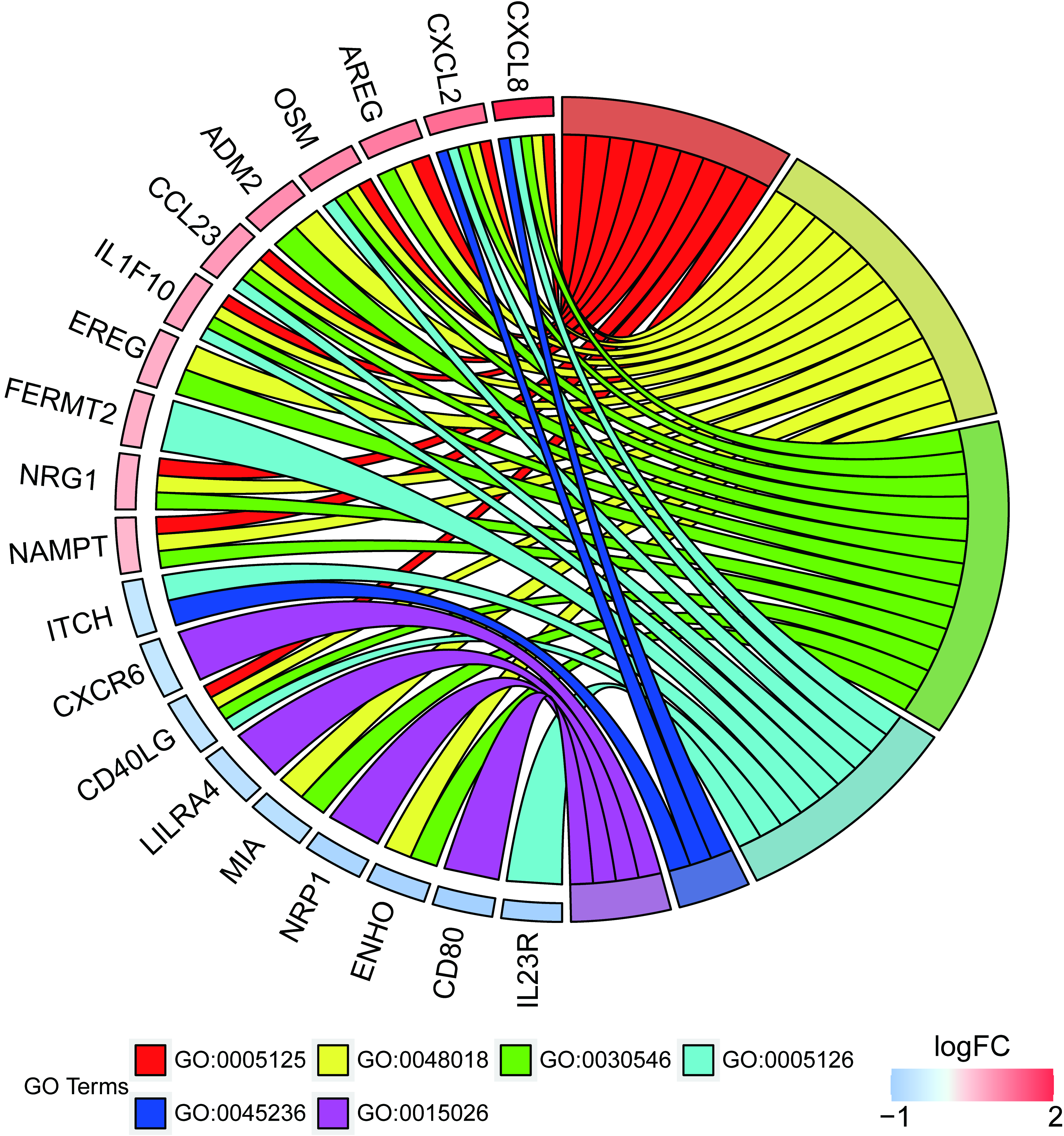

Supplement: Supplementary file 1 [file ijms-24-10619-s001.zip › Supplementary figures/Figure S2.tif]

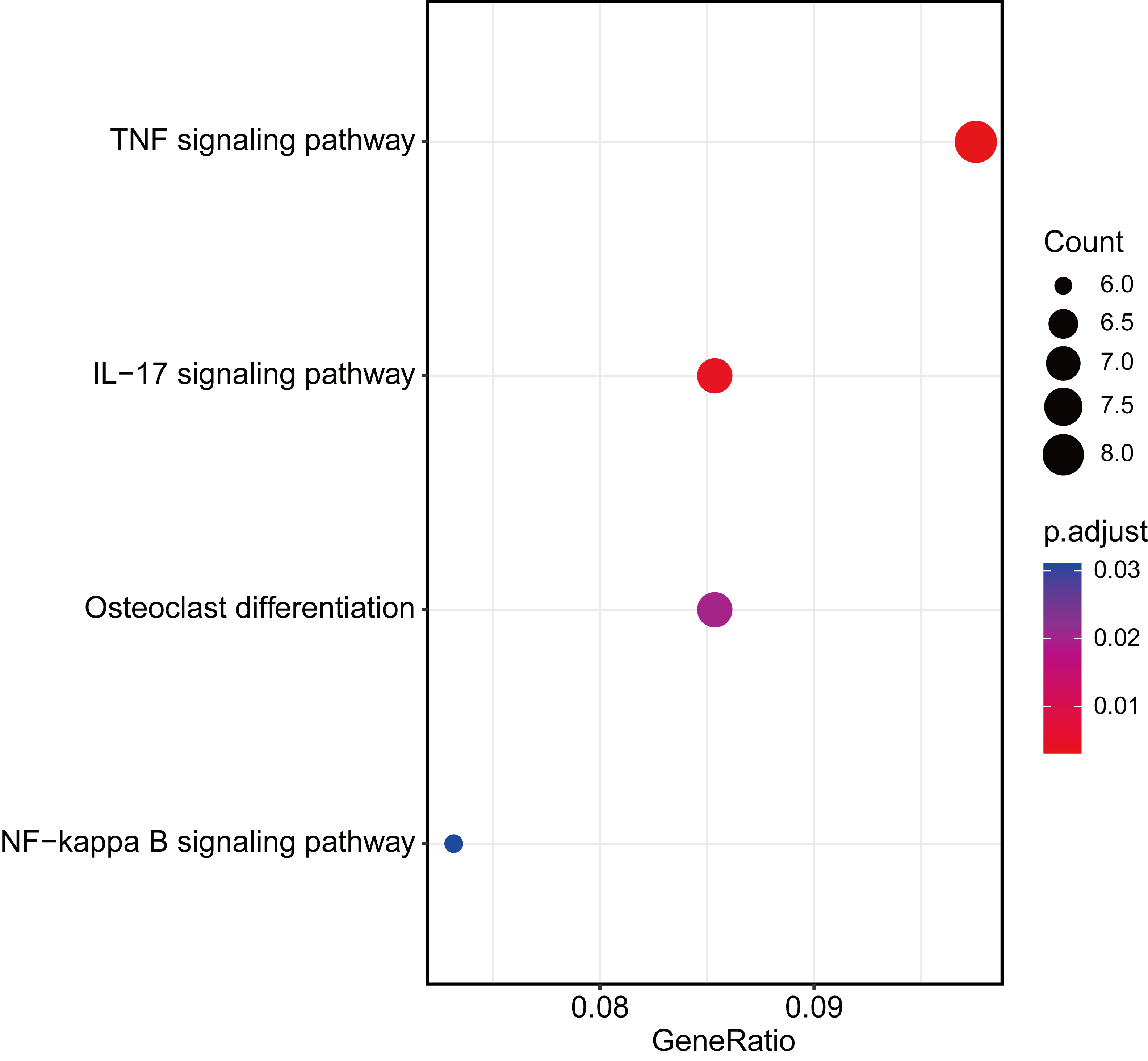

Supplement: Supplementary file 1 [file ijms-24-10619-s001.zip › Supplementary figures/Figure S3.tif]

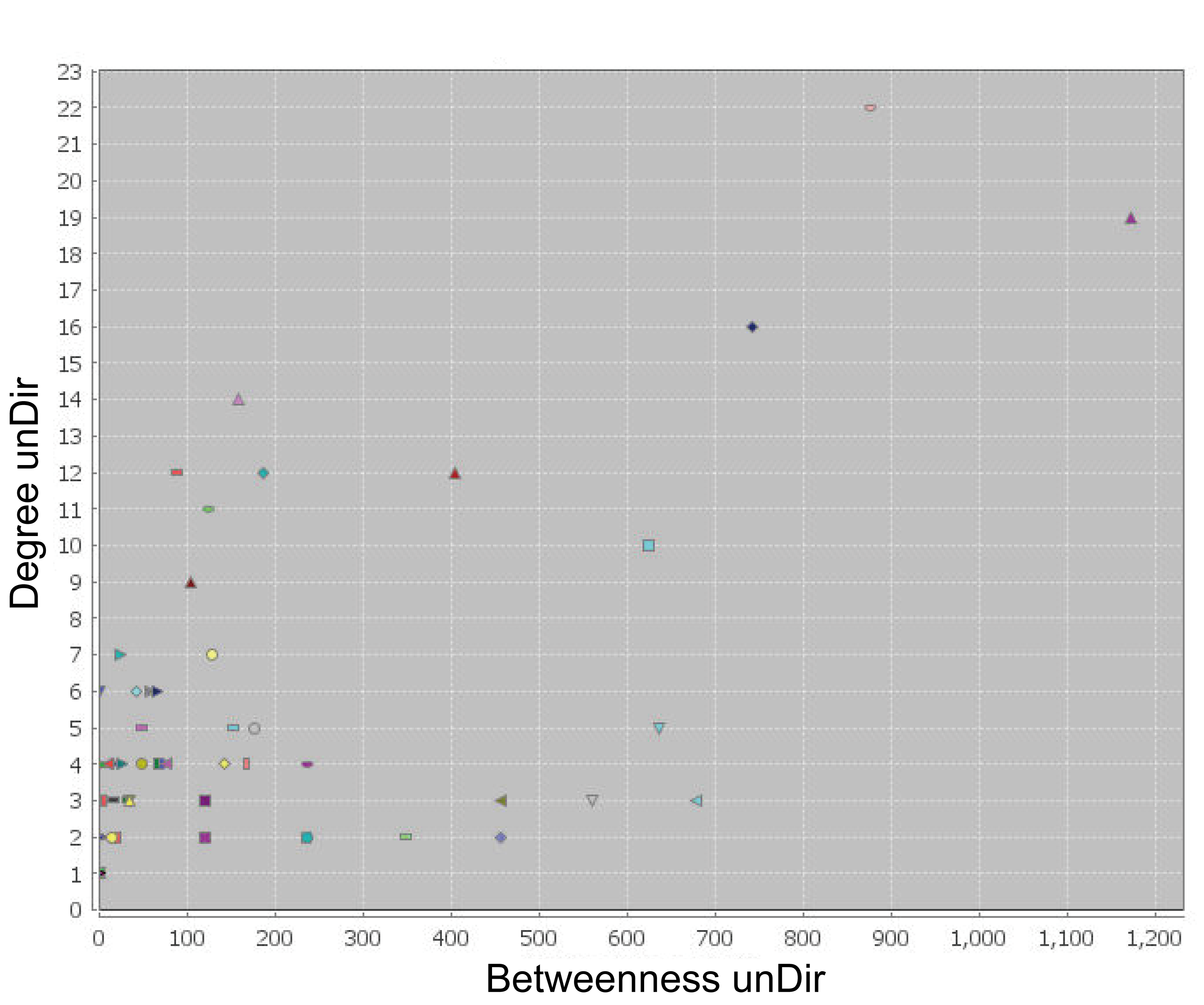

Supplement: Supplementary file 1 [file ijms-24-10619-s001.zip › Supplementary figures/Figure S4.tif]

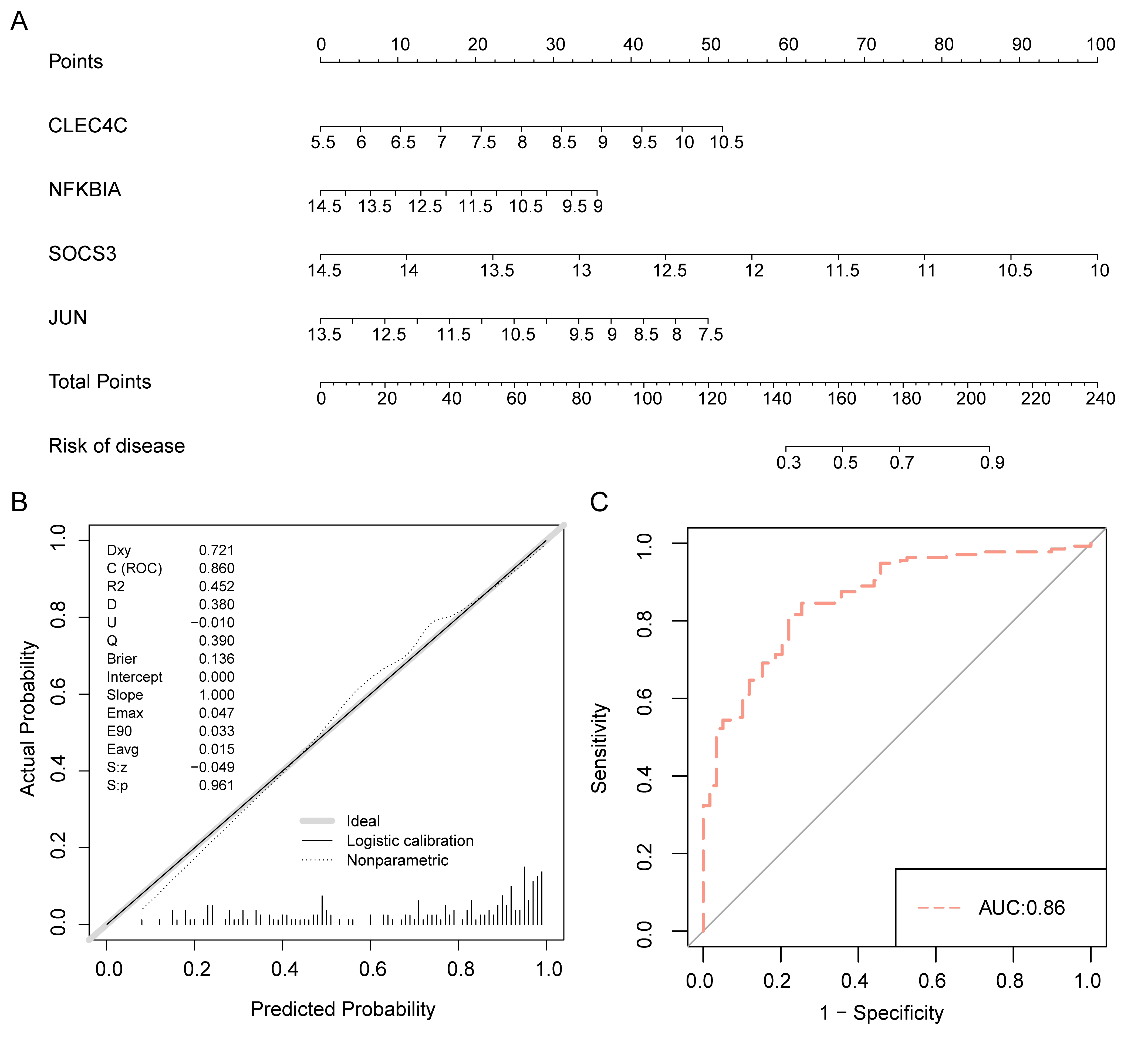

Supplement: Supplementary file 1 [file ijms-24-10619-s001.zip › Supplementary figures/Figure S5.tif]

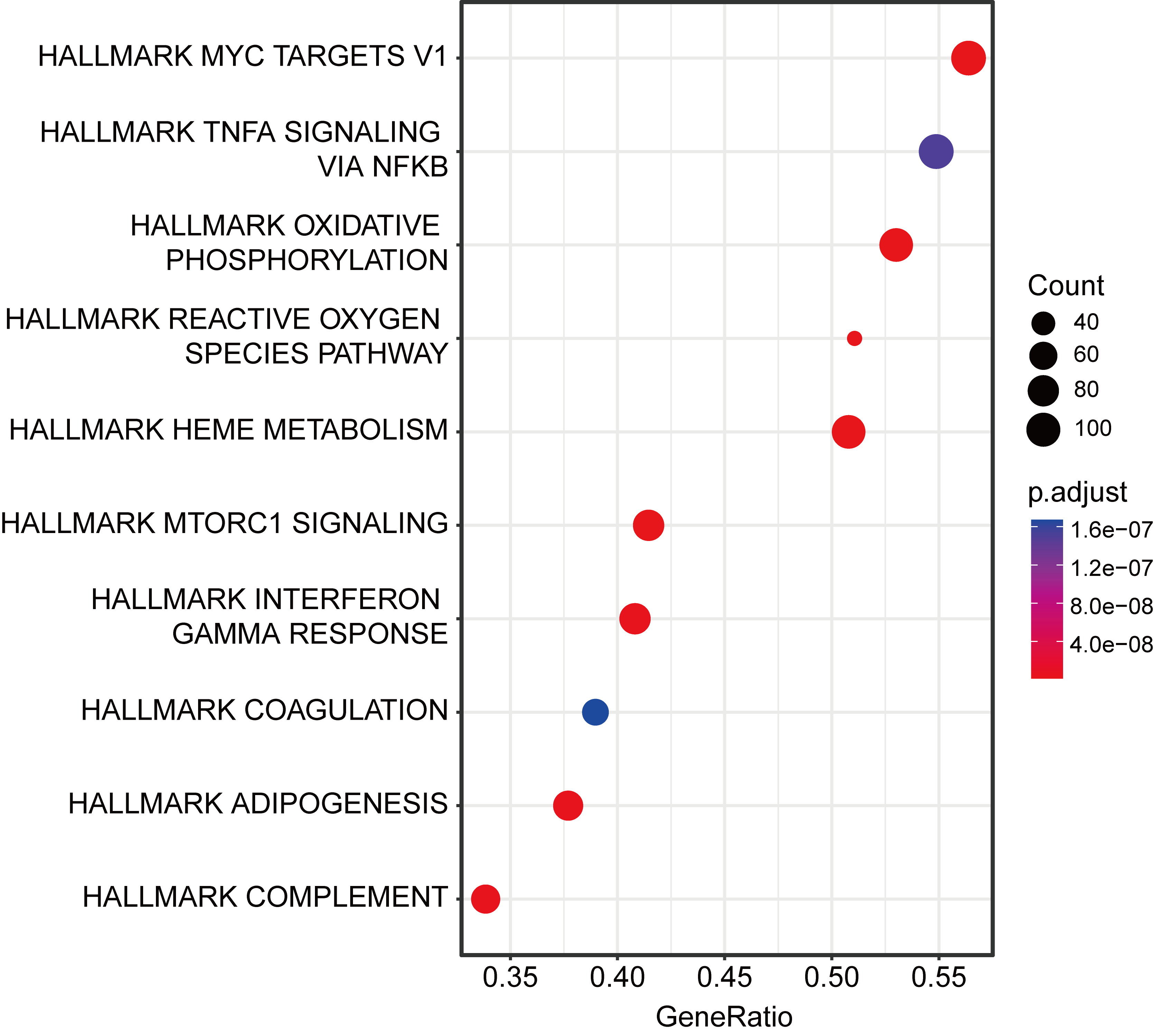

Supplement: Supplementary file 1 [file ijms-24-10619-s001.zip › Supplementary figures/Figure S6.tif]

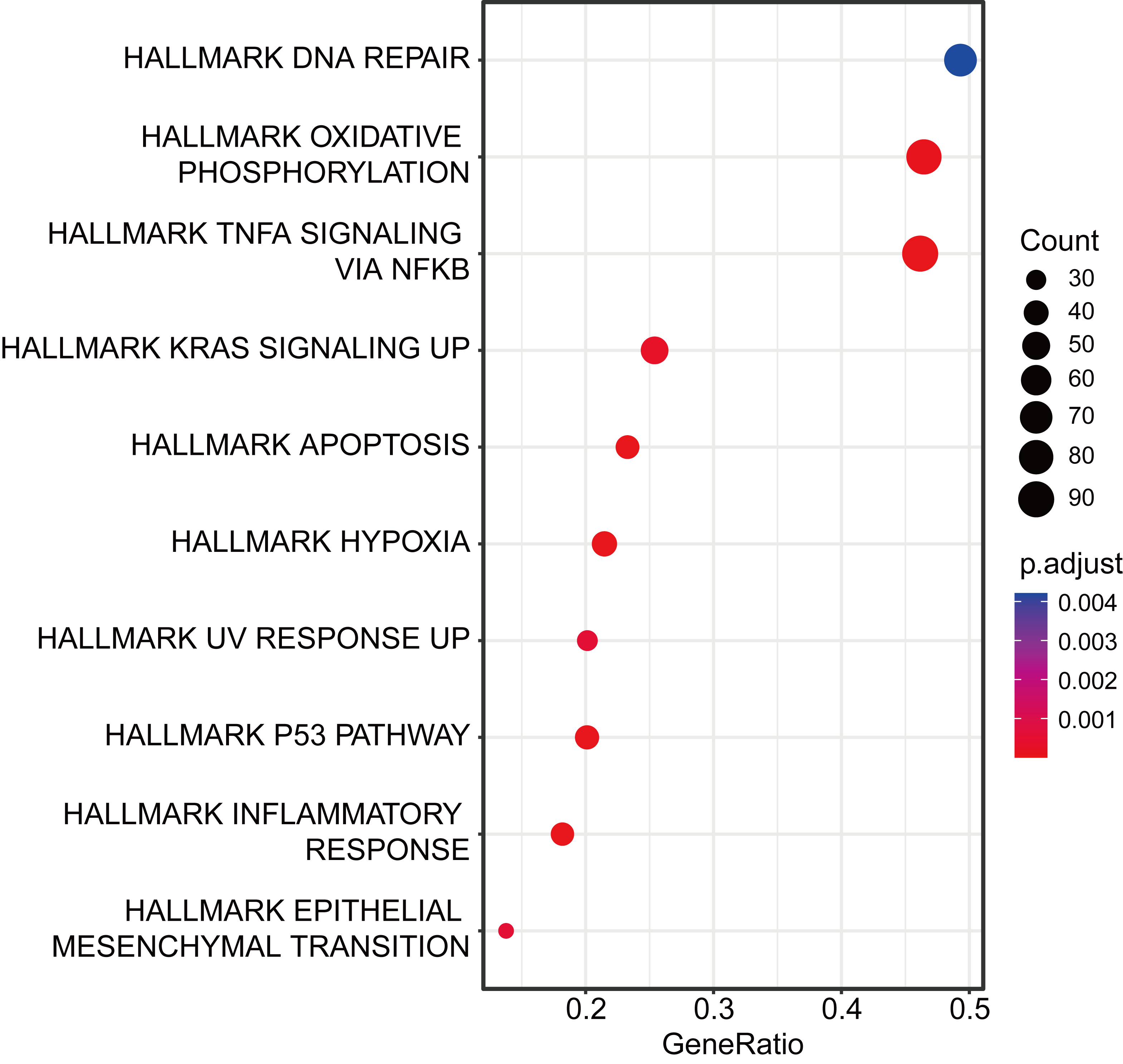

Supplement: Supplementary file 1 [file ijms-24-10619-s001.zip › Supplementary figures/Figure S7.tif]

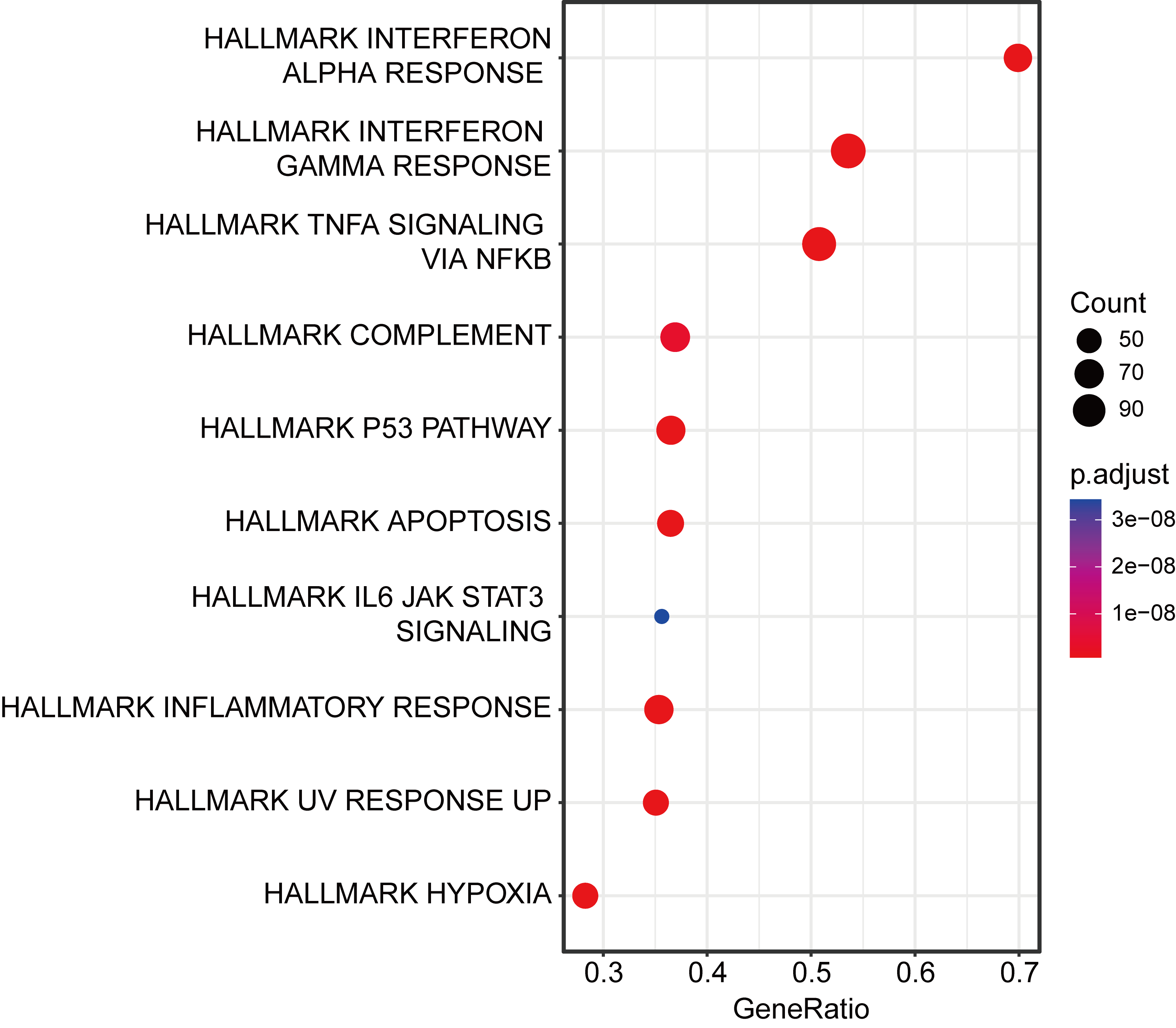

Supplement: Supplementary file 1 [file ijms-24-10619-s001.zip › Supplementary figures/Figure S8.tif]

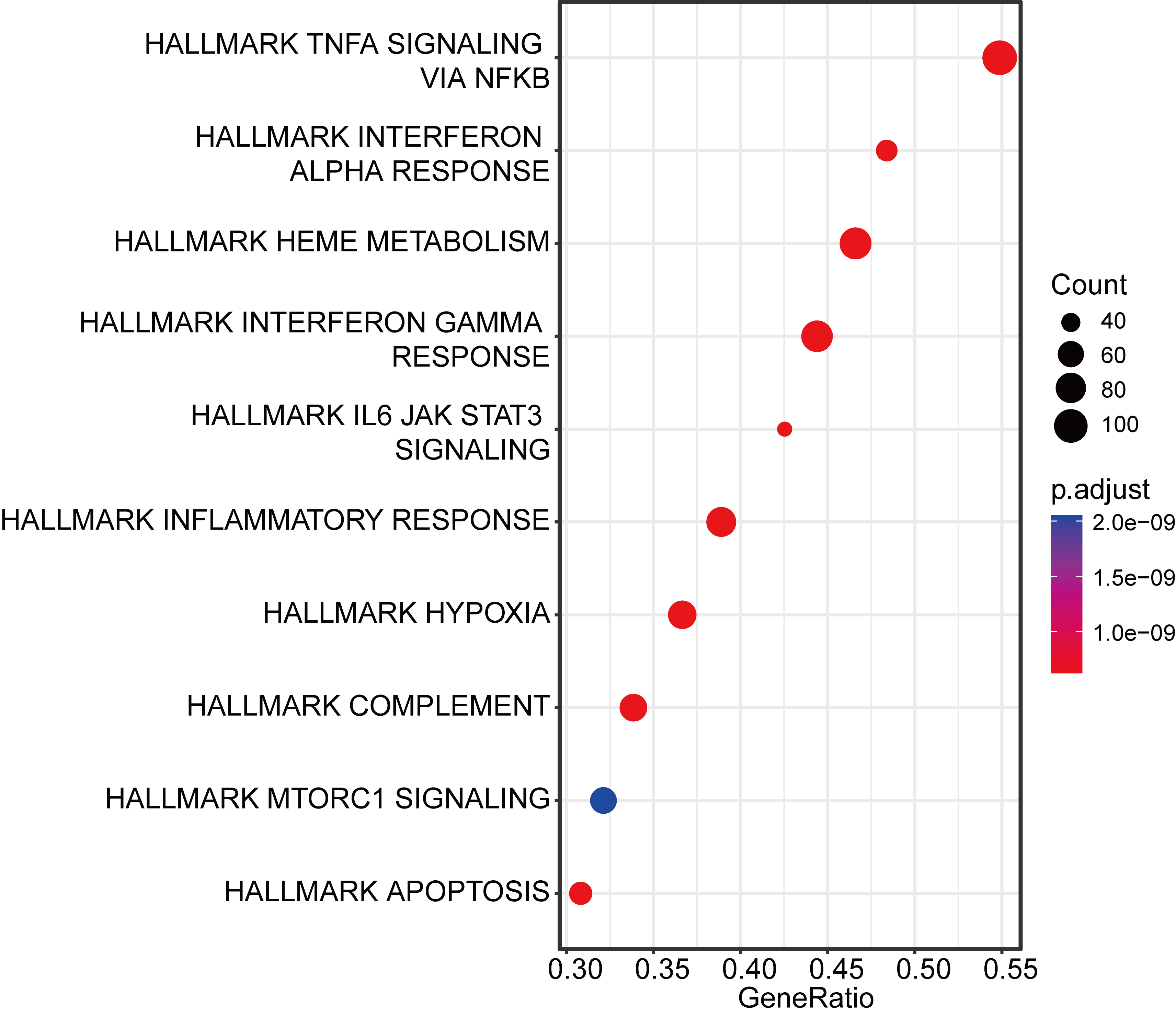

Supplement: Supplementary file 1 [file ijms-24-10619-s001.zip › Supplementary figures/Figure S9.tif]
